# Pingkui Enema Alleviates TNBS-Induced Ulcerative Colitis by Regulation of Inflammatory Factors, Gut Bifidobacterium, and Intestinal Mucosal Barrier in Rats

**DOI:** 10.1155/2020/3896948

**Published:** 2020-08-05

**Authors:** Hai-Feng Yun, Rui Liu, Dan Han, Xin Zhao, Jin-Wei Guo, Feng-Jiao Yan, Chuan Zhang, Hong-Wen Sun, Guo-Qiang Liang, Guo-Xing Zhang

**Affiliations:** ^1^Department of Internal Medicine, Suzhou Traditional Chinese Medicine Hospital Affiliated to Nanjing University of Chinese Medicine, 18 Yang-Su Road, Suzhou 215003, China; ^2^Suzhou Academy of Wumen Chinese Medicine, Suzhou Traditional Chinese Medicine Hospital Affiliated to Nanjing University of Chinese Medicine, 18 Yang-Su Road, Suzhou 215003, China; ^3^Department of Physiology, Medical College of Soochow University, 199 Ren-Ai Road, Dushu Lake Campus, Suzhou Industrial Park, Suzhou 215123, China

## Abstract

**Background:**

Ulcerative colitis (UC) is a chronic recurrent inflammation of the colon, and clinical outcome of UC is still unsatisfied. Pingkui enema, a traditional Chinese medicine prescription, has been safely applied for the treatment of diarrhea and dysentery in clinic for many years. However, its mechanism is still elusive. The present study is designed to investigate the effect of Pingkui enema on trinitrobenzene sulfonic acid- (TNBS-) induced ulcerative colitis (UC) and possible mechanism in rats.

**Methods:**

UC was induced by intracolonic instillation of TNBS in male Sprague-Dawley rats, which were treated with different dosages of Pingkui enema (low, medium, and high) or sulfasalazine for ten days. Survival rate was calculated. A clinical disease activity score was evaluated. Histological colitis severity was analyzed by hematoxylin-eosin (HE) staining. Content of Bifidobacterium in intestinal tissue was analyzed by RT-PCR. Concentration of IL-8, IL-13, TNF-*α*, *D*-lactic acid (*D*-LA), and diamine oxidase (DAO) in serum and contents of adhesin and receptor of Bifidobacterium adhesion in rat intestinal mucus were measured by ELISA.

**Results:**

The results showed that Pingkui enema treatment with high dosage markedly improved the survival rate compared with untreated and sulfasalazine treated groups. All dosages of Pingkui enema reduced pathological score. High dosage of Pingkui enema and sulfasalazine treatments significantly reduced the serum concentration of IL-8, TNF-*α*, *D*-LA, and DAO and markedly increased the serum concentration of IL-13. In addition, high-dose Pingkui enema and sulfasalazine treatments increased gut content of Bifidobacterium, gut mucus expressions of adhesin, and adhesin receptor of Bifidobacterium.

**Conclusions:**

Pingkui enema has therapeutic effect on TNBS-induced UC, and possible mechanism may be via regulation of gut probiotics (Bifidobacterium) and inflammatory factors and protection of intestinal mucosal barrier.

## 1. Backgrounds

Ulcerative colitis (UC) is a kind of immune system disease whose etiology is not completely clear. The clinical manifestations are diarrhea, mucous pus and bloody stool, abdominal pain, and tenesmus. Data analysis showed that, after 8–10 years of UC onset, the risk of incidence of cancer increased by 0.5%–1.0% annually, and the probabilities of incidence of cancer were 2%, 8%, and 18% with 10, 20, and 30 years of UC course, respectively [1]. Nowadays, mesalazine, 5-aminosalicylates (5-ASA), and sulfasalazine (SASP) are the main drugs for the treatment of mild to moderate UC [2]. The short-term curative effect of these drugs on UC is certain, but it is easy to recur and the symptoms are repeated [3]. In addition, most of these drugs have limitations in side effects and safety, such as drug dependence, adverse reaction, decline of immune function, and risk of cancer [4–6]. Therefore, it is urgent to explore novel strategy for treating UC.

Relevant studies have shown that many cytokines, such as TNF-*α*, IL-8, and IL-13, are involved in the occurrence and development of inflammatory reaction in intestinal mucosa in the UC model [7]. Anti-TNF-*α* agents have shown a significant advance in the management of ulcerative colitis [8]. IL-8 antagonist coupled with probiotics exhibits variably enhanced therapeutic potential in ameliorating UC [9]. IL-13 in colonic mucosa of active UC patients was significantly higher than that of inactive UC patients [10, 11]. Targeting on inflammatory cytokine is one of the strategies on treatment of UC.

Epithelial barrier defect is one of the pathogenesis of UC [2]. The serum levels of diamine oxidase (DAO) and *D*-lactic acid (*D*-LA) are important biochemical markers for evaluating epithelial barrier function. UC models have been reported to increase serum levels of DAO and *D*-LA [12, 13], suggesting there is a defection of epithelial barrier in UC. Clinical data also supported that there are high serum levels of DAO and *D*-LA in UC patients [14].

UC is associated with a substantial alteration of specific gut commensals, some of which may be involved in microbiota-mediated protection. Bifidobacterium is reported as novel microbial biomarkers in UC [15]. Clinical trial treatment with Bifidobacterium longum 536 showed beneficial effects on UC patients [16]. It is also observed that pathogenic *E coli* is involved in development of UC, and pathogenic *E coli* has adhesive properties which are mirrored by an increase in their surface hydrophobicity. *E coli* isolated from patients with ulcerative colitis possess a mannose-resistant adhesin similar to that found in pathogenic *E coli* [17, 18]. Therefore, it is important to regulate gut probiotics and pathogenic bacteria in UC patients; however, there is a rare literature reporting the effects of TCM on regulation of gut bacteria.

Recently, many studies reported that TCM has a remarkable effect on UC animal model and patients, and its effect on these inflammatory factors has also been reported [19–21]. Pingkui enema is a TCM herb compound prescription, which was firstly recorded in TCM classic medical work “Treatise on Cold Pathogenic Diseases” by Zhang Zhong-Jing (A.D. 150–215). The prescription is widely used in patients with diarrhea, UC, and other intestinal diseases. Nowadays, Pingkui enema is developed based on previous four herbs: Radix Pulsatillae (*Pulsatilla chinensis* (Bunge) Regel), Rhizoma Coptidis (*Coptis chinensis* Franch.), Phellodendri Chinensis Cortex (*Phellodendron amurense* Rupr.), and Fraxini Cortex (*Fraxinus rhynchophylla* Hance), by adding another four herbs: Hairyvein Agrimony (*Agrimonia eupatoria* L.), Panax Notoginseng (*Panax pseudoginseng* var. *notoginseng* (Burkill) G.Hoo and C.L.Tseng), Radix Arnebiae (*Lithospermum officinale* var. *Erythrorhizon* (Siebold and Zucc.) Maxim.), and Rhizoma Bletillae (*Bletilla striata* (Thunb.) Rchb. f.). All of these herbs had few side effects and low costs and had been safely used in clinic for UC patients [22–24]. However, effects and mechanisms of Pingkui enema on UC are still elusive.

In present study, the UC rat model was established by trinitrobenzene sulfonic acid (TNBS) as previous reported [25]. The serum levels of IL-8, IL-13, TNF-*α*, *D*-LA, and DAO, the intestinal feces content of Bifidobacterium, and colonization ability of Bifidobacterium were detected. The effects and possible mechanisms of Pingkui enema on UC rats were investigated.

## 2. Methods

### 2.1. Preparation of Pingkui Enema

Pingkui enema is provided by Pharmaceutical Department of Suzhou Traditional Chinese Medicine Hospital. According to the conventional decocting method of the standard of Chinese Pharmacopoeia (2015 edition), eight herbs (Table 1) were washed, mixed, and extracted by distilled water two times. The extracts were evaporated under reduced pressure and constant volume to equivalent 2 g/mL of crude drug and stored in a refrigerator at 4 C.

### 2.2. Experimental Animals

Sixty Sprague-Dawley rats were provided by Zhaoyan (Suzhou) New Drug Research Center Co., Ltd. half male and half female, weighing 200 ± 20 g. The approval number of animal experiment of this study was SCXK (Su) 2013–0015. Rats were routinely raised in SPF animal room of Suzhou Institute of Traditional Chinese Medicine Hospital. The experiments were performed in accordance with the National Institutes of Health Guidelines for the Use of Laboratory Animals (NIH, publication number 85–23, revised 1996), which were approved by and performed according to guidelines for the care and use of animals established by Suzhou Institute of Traditional Chinese Medicine Hospital.

Sixty rats were randomly divided to six groups: (1) control group (*n* = 8), (2) UC group (*n* = 12), (3) UC plus low dose of Pingkui enema (*n* = 10), (4) UC plus medium dose of Pingkui enema (*n* = 10), (5) UC plus high dose of Pingkui enema (*n* = 10), and (6) UC plus sulfasalazine enteric-coated tablets (*n* = 10). The UC model was induced by 2,4,6-trinitrobenzene sulfonic acid (TNBS, batch number 2508-19-2) according to literature [25]. TNBS was slowly injected by a 1 mL syringe connected silicone tube, from rats anal, and depth of tube from anal is 8 cm. Rats were fixed anatomically at 45° for 10 min to ensure good contact of drug with rat colon lumen. The administration style of Pingkui enema was same with TNBS, started on the 4th day after the TNBS treatment, and Pingkui enema was diluted in proportion with distilled water and heated to 35°C–37°C for enema (according to the algorithm of body surface area conversion, the dosage of Pingkui enema in low, medium, and high groups was 4.32, 8.64, and 17.25 g/kg, respectively). Sulfasalazine group was given 0.27 g/kg sulfasalazine (calculation of equivalent dose ratio 0.018 based on the body surface area of human and rat), which was purchased from Shanghai Fuda Pharmaceutical Co., Ltd. (batch no. 22170102). Disease activity index was evaluated according to Table 2. After the last administration, rats were fasted for 24 hours and sacrificed under sodium pentobarbital (50 mg/kg i.p.) anesthesia. Rats serum was collected for further examination. The colon was taken up 2 cm from the anus by laparotomy. The colon was cut longitudinally and washed with distilled water. Parts of samples were fixed with 10% formalin and embedded in paraffin after 24 hours for further analysis. Parts of animal samples were used to collect mucus on the mucosa surface after washing with 0.01 mol/L sterile phosphate buffer (pH = 7.5 ± 0.1) for the following detection.

### 2.3. Histopathological Observation

The paraffin sections were cut into 4 *μ*m thickness with the microtome and then were stained with hematoxylin-eosin (HE). The histological scores were calculated according to the damages of inflammatory infiltration and epithelium mucosa. The standard for pathological scoring is shown in Table 3 [19].

### 2.4. Detection of the Content of Adhesin and Adhesin Receptor of Bifidobacterium in Intestinal Mucus

Collected mucus samples were centrifuged with 3000 r/min for 10 minutes at 4°C, and supernatants were used to detect Bifidobacterium adhesion and adhesin receptor by enzyme-linked immunosorbent assay (ELISA) kit (Shanghai Bangyi Biotechnology Co., Ltd., batch no.: bye21023; bye21029).

### 2.5. Real-Time Fluorescence Quantitative PCR Was Used to Detect the Content of Bifidobacterium in Colon Tissue

The feces of rat were collected from the cecum. Genomic QIAamp DNA Stool Mini Kit was used to extract genomic DNA from feces samples. Real-time PCR quantitative detection kit of Bifidobacterium adolescentis gene (Micro-Based Biotechnology Co., Ltd.) was used for fast qPCR. The forward primers BiADOg-1a and BiADOg-1b were 5′-CTCCAGTTGGATGCATGTC-3′, 5′-TCCAGTTGACCGCATGGT-3'. Real-time quantitative PCR was performed in FTC-3000 fluorescent quantitative PCR instrument. All samples were repeated 3 times.

### 2.6. Detecting the Serum Levels of IL-8, IL-13, TNF-*α*, D-LA, and MAO in Rats

The serum levels of IL-8, IL-13, TNF-*α* (Shanghai Bangyi Biotechnology Co., Ltd., batch number: bye20700; bye20039; bye20040), *D*-LA, and MAO (Sigma, batch number: RAB0411-1KT, RAB0028-1KT) were measured by ELISA kits according to the manufacture's instruction.

### 2.7. Statistical Analysis

One-way ANOVA was used for comparison between groups followed by Turkey analysis. Chi-square test was used for counting data. *P* < 0.05 was considered to statistical significance.

## 3. Results

### 3.1. Rat Survival Rate

**Figure 1:**
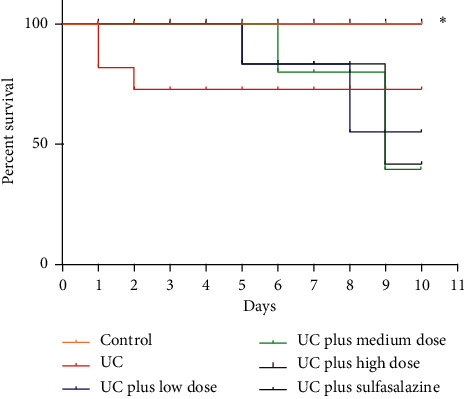
Survival rate of rats in each group. Control indicates the control group (*n* = 8), UC indicates the UC group (*n* = 12), UC plus low dose indicates UC plus low dose of Pingkui enema (*n* = 10), UC plus medium dose indicates UC plus medium dose of Pingkui enema (*n* = 10), UC plus high dose indicates UC plus high dose of Pingkui enema (*n* = 10), and UC plus sulfasalazine indicates UC plus sulfasalazine enteric-coated tablets (*n* = 10). ^*∗*^*P* < 0.01 compared with the UC group and UC plus sulfasalazine group.

Three rats in the UC group died during the experiment. After administration Pingkui enema, the survival rate in high dosage of Pingkui enema group was markedly improved (*P* < 0.01 compared with the UC group), and it was also significantly better than the sulfasalazine group (*P* < 0.01 compared with UC plus sulfasalazine group) (Figure 1).

### 3.2. Body Weight and DAI

**Figure 2:**
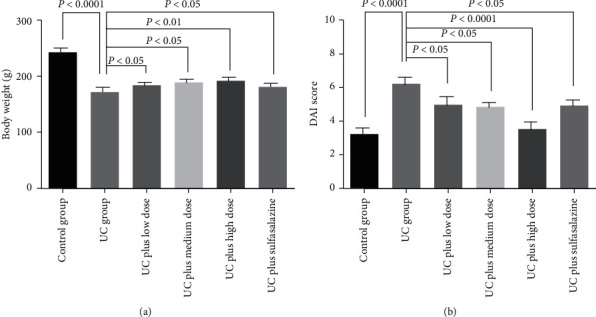
(a) Body weight and (b) DAI score in each group. Control indicates the control group (*n* = 8), UC indicates the UC group (*n* = 12), UC plus low dose indicates UC plus low dose of Pingkui enema (*n* = 10), UC plus medium dose indicates UC plus medium dose of Pingkui enema (*n* = 10), UC plus high dose indicates UC plus high dose of Pingkui enema (*n* = 10), and UC plus sulfasalazine indicates UC plus sulfasalazine enteric-coated tablets (*n* = 10).

The body weight of rat was lower in the TNBS-induced UC group after 10 days compared with the control group. All dosages of Pingkui enema treatments showed less reduction of body weight compared with the UC group. Sulfasalazine treatment also showed the similar effect on body weight reduction with Pingkui enema (Figure 2(a)).

The DAI score in the UC group was markedly higher compared with the control group. All dosages of Pingkui enema treatments reduced DAI score compared with the UC group. Sulfasalazine treatment also reduced DAI score compared with the UC group. In addition, all dosages of Pingkui enema treatments significantly alleviated synechia, edema, and hyperemia induced by TNBS (data not shown).

### 3.3. Histopathological Observation

**Figure 3:**
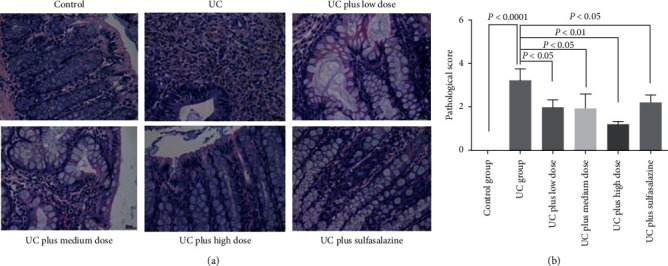
Histopathological analysis of rat colon tissue: (a) HE staining of colon tissue in each group (*n* = 5); (b) pathological score of colon tissue in each group (*n* = 5). Control indicates the control group, UC indicates the UC group, UC plus low dose indicates UC plus low dose of Pingkui enema, UC plus medium dose indicates UC plus medium dose of Pingkui enema, UC plus high dose indicates UC plus high dose of Pingkui enema, and UC plus sulfasalazine indicates UC plus sulfasalazine enteric-coated tablets.

TNBS induced severe inflammation, cell infiltration, and crypt loss, consistent with the DAI scores (Figure 2(b)). The inflammatory response in the colons of rats in the all dosages of Pingkui enema groups and the sulfasalazine group showed a distinct decrease in these changes compared with the colons of rats in the TNBS-induced UC group (Figures 3(a) and 3(b)).

### 3.4. Serum Levels of IL-8, IL-13, TNF-*α*, *D*-LA, and MAO in Rats

**Figure 4:**
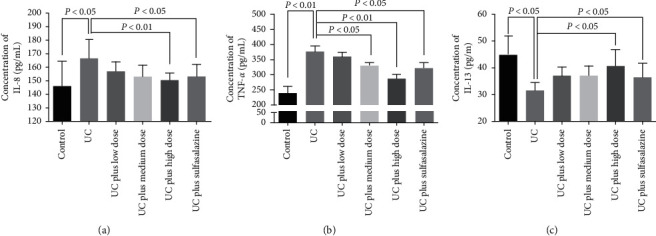
Serum levels of IL-8, TNF-*α*, and IL-13 in rats: (a) concentration of IL-8 in serum of rats in each group (*n* = 7–10); (b) concentration of TNF-*α* in serum of rats in each group (*n* = 7–10); (c) concentration of IL-13 in serum of rats in each group (*n* = 7–10). Control indicates the control group, UC indicates the UC group, UC plus low dose indicates UC plus low dose of Pingkui enema, UC plus medium dose indicates UC plus medium dose of Pingkui enema, UC plus high dose indicates UC plus high dose of Pingkui enema, and UC plus sulfasalazine indicates UC plus sulfasalazine enteric-coated tablets.

Serum inflammatory factors IL-8, IL-13, and TNF-*α* were detected by ELISA. As shown in Figure 3, the UC group showed increase in serum levels of IL-8 (Figure 4(a)) and TNF-*α* (Figure 4(b)) and decrease in serum levels of IL-13 (Figure 4(c)); treatments with high dose of Pingkui enema and sulfasalazine could reverse these inflammatory factors expression in serum (Figures 4(a)–4(c)).

**Figure 5:**
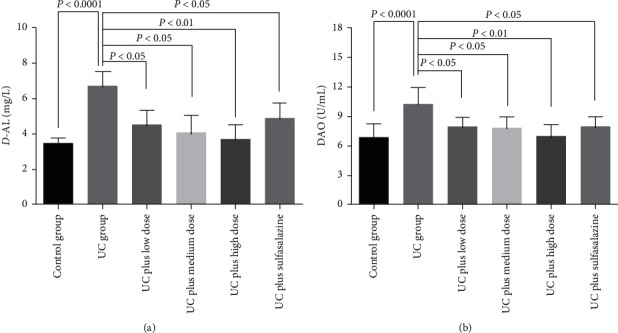
Serum levels of D-AL and DAO in rats: (a) concentration of D-AL in serum of rats in each group (*n* = 7–10); (b) concentration of DAO in serum of rats in each group (*n* = 7–10). Control indicates the control group, UC indicates the UC group, UC plus low dose indicates UC plus low dose of Pingkui enema, UC plus medium dose indicates UC plus medium dose of Pingkui enema, UC plus high dose indicates UC plus high dose of Pingkui enema, and UC plus sulfasalazine indicates UC plus sulfasalazine enteric-coated tablets.

Indexes of intestinal epithelial barrier function and serum levels of *D*-AL and DAO were also detected by ELISA. As shown in Figure 5, UC group showed increase in serum levels of *D*-AL (Figure 5(a)) and DAO (Figure 5(b)). Treatments with all dosages of Pingkui enema and sulfasalazine reduced serum levels of *D*-AL and DAO (Figures 5(a) and 5(b)).

### 3.5. Adhesin and Adhesin Receptor Levels of Bifidobacterium Intestinalis in Rats

**Figure 6:**
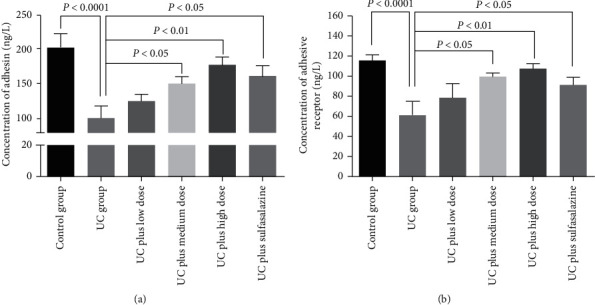
Adhesin and adhesin receptor levels of Bifidobacterium intestinalis in rat intestinal mucosa: (a) levels of adhesin in each group (*n* = 7–10); (b) Bifidobacterium adhesin receptor content in each group (*n* = 7–10). Control indicates the control group, UC indicates the UC group, UC plus low dose indicates UC plus low dose of Pingkui enema, UC plus medium dose indicates UC plus medium dose of Pingkui enema, UC plus high dose indicates UC plus high dose of Pingkui enema, and UC plus sulfasalazine indicates UC plus sulfasalazine enteric-coated tablets.

Adhesin and adhesin receptor levels of Bifidobacterium intestinalis in rat intestinal mucosa were detected by ELISA. As shown in Figure 6, Bifidobacterium adhesin (Figure 6(a)) and adhesin receptor (Figure 6(b)) in the UC group were significantly decreased compared with the control group; medium and high dosages of Pingkui enema and sulfasalazine treatments could increase levels of Bifidobacterium adhesin and adhesin receptor (Figure 6).

### 3.6. Relative Content of Bifidobacterium in Intestinal Mucosa of Rats

**Figure 7:**
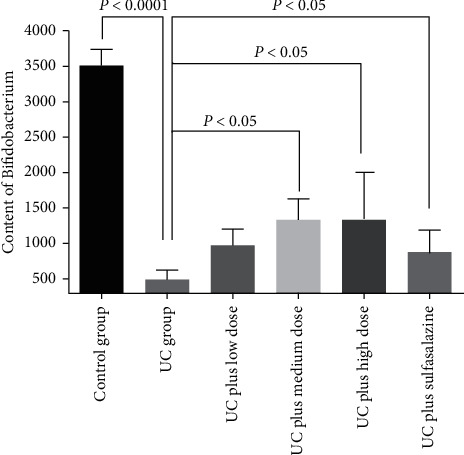
Content of Bifidobacterium in colon tissue of rats (*n* = 3). Control indicates the control group, UC indicates the UC group, UC plus low dose indicates UC plus low dose of Pingkui enema, UC plus medium dose indicates UC plus medium dose of Pingkui enema, UC plus high dose indicates UC plus high dose of Pingkui enema, and UC plus sulfasalazine indicates UC plus sulfasalazine enteric-coated tablets.

Fluorescence quantitative PCR was used to detect the change of Bifidobacterium content in colon stool of rats in each group. The results showed that the content of Bifidobacterium in intestinal mucosa of rats in UC group was significantly decreased compared with control group, the medium dose Pingkui enema treatment increased content of Bifidobacterium in intestinal mucosa of rats (Figure 7).

## 4. Discussion

In the present study, our observation reveals that Pingkui enema has therapeutic effect on TNBS-induced UC, and a possible mechanism may be via regulation of gut probiotics (Bifidobacterium) and inflammatory factors and protection of intestinal mucosal barrier.

UC is a multifactorial nonspecific intestinal inflammation characterized by diffuse distribution in the colon, the most common site of which involves sigmoid colon and rectum. Epidemiological investigation showed that the predisposing age of UC was 20–50-year-old, and there was no gender difference [26]. The incidence of UC is increasing year by year and has been recognized as a refractory disease by the World Health Organization. Its pathogenesis and development are related to immune factors, genetic factors, dietary factors, infectious factors, mental factors, and other factors. Probiotics can be colonized in the intestinal tract of animals, maintaining the balance of intestinal flora, playing an important role in intestinal mucosal immunity, including Bifidobacterium, Lactobacillus, and *Streptococcus*. A large number of studies have shown that probiotics can improve the intestinal microenvironment, prolong the remission period of inflammation, and reduce the incidence of UC-related colon cancer [27, 28]. Study has shown that intestinal flora imbalance is one of the pathogenesis of UC [29]. It also has been reported that contents of Bifidobacterium, Lactobacillus, *Bacillus fragilis,* and *Clostridium* were decreased, and contents of *Bacillus difficile*, *Bacillus* pseudobacteria, Botulinum toxin, *Clostridium,* and *Enterococcus faecalis* were increased in the intestine of the rat UC model [30, 31]. At present, probiotics used to treat UC mainly include Lactobacillus, Bifidobacterium, and various mixed symbiotic bacteria. The possible mechanisms of probiotics in the treatment of UC include competitive rejection of pathogenic bacteria, reduction of intestinal inflammatory response, and regulation of intestinal mucosal immune function. Bifidobacterium can form a protective biofilm on the surface of intestinal mucosa by binding specifically to intestinal epithelial cells and inhibit the growth and invasion of anomalous aerobic flora. Colonizing on the intestinal epithelial is prerequisite for Bifidobacterium plays its protective role, which mainly depends on the specific binding of adhesin and its receptor. Our present observation clearly demonstrated that Bifidobacterium content and its colonization ability were decreased by TNBS. These results support there is a flora imbalance in TNBS-induced UC [29].

According to theory of TCM from “Treatise on Cold Pathogenic Diseases,” UC patients show tenesmus symptom due to the hot inside the body, while *Radix Pulsatillae* belongs to the bitter and cold herb and could be mainly applied for against hot in UC patients, therefore exerting therapeutic effect. The Pingkui enema used in the present study is modified from previous classic prescription “decoction of *Radix Pulsatillae*” from “Treatise on Cold Pathogenic Diseases.” The main herb of “decoction of *Radix Pulsatillae*” is *Radix Pulsatillae*, other three herbs are *Phellodendri Chinensis Cortex, Rhizoma Coptidis*, and *Fraxini Cortex* as complementary herbs, also has effects on decrease of hot detoxification according to theory of TCM pharmacology. Based on modern study, this four herbs also showed therapeutic properties on UC, *Radix Pulsatillae* has anti-inflammatory and immune-modulatory properties [32], *Fraxini Cortex* has obvious anti-inflammatory and analgesic effects [33], and berberine extracted from *Rhizoma Coptidis* and *Phellodendri Chinensis Cortex* can significantly inhibit the opening of delayed rectifier potassium channels and Ca^2+^ activated potassium channels on the surface of guinea pig colonic smooth muscle cells, therefore, inhibit the contraction of colonic smooth muscle, and be used to treat abdomen diarrhea [34]. Besides the abovementioned four classical herbs, four other herbs were supplemented in Pingkui enema based on traditional TCM theory and recent observations. *Hairyvein Agrimony* has analgesic, anti-inflammatory, and hemostatic effects [35]. *Panax Notoginseng* has been reported to regulate intestinal microbiota [36]. *Radix Arnebiae* can effectively inhibit the LPS/rIFN-gamma-induced production of NO and TNF-alpha [37]. *Rhizoma Bletillae* not only has been widely used for the treatment of hematemesis, hemoptysis, and traumatic bleeding but also has been applied topically to overcome ulcers, sores, swellings, and chapped skin due to the efficacy of dispersing swelling and promoting tissue regeneration [38]. It should be noted that clinical applicant of Pingkui enema showed satisfactory outcome in UC patients, supporting the advancement for the modification of classic prescription. For further servicing the clinical patients, it is necessary to fully clarify the mechanisms of Pingkui enema on UC patients.

Our present observation demonstrated that Pingkui enema alleviated the UC symptoms induced by TNBS (increase in body weight and DAI) in rats. To explore the possible mechanisms, we firstly investigated effects of Pingkui enema on intestinal inflammation, one of the common pathogenesis in UC. Markers of inflammation of serum levels of IL-8, IL-13 and TNF-*α* were examined. Our data showed that the serum levels of IL-8 and TNF-*α* in the Pingkui enema group were decreased significantly. These results demonstrated that inhibition of inflammatory cytokines may be one of the mechanisms of therapeutic effects of Pingkui enema on TNBS-induced UC. However, our result also showed that level of IL-13 was increased significantly after Pingkui enema treatment although IL-13 is regarded as a marker of active UC condition, and it also has been demonstrated that IL-13 might be not essential in the development of inflammatory bowel disease [39]. At present, we still have no good explanation for the discrepancy of decrease in IL-13 in response to TNBS treatment and increase in IL-13 level by Pingkui enema after TNBS treatment, and we speculate that IL-13 might act as an anti-inflammatory cytokine in TNBS-induced UC since our data showed that TNBS treatment decreased IL-13 levels. Further observation is still needed to clarify this issue. Secondly, we explored the possible effects of Pingkui enema on regulating the balance of intestinal microbiota. We focused on one of the probiotics, Bifidobacterium. Content of Bifidobacterium in stool and expressions of adhesin and adhesin receptor were analyzed. Our data showed Pingkui enema significantly increased Bifidobacterium mRNA in rat stool, accompanied with elevation of adhesin and adhesin receptor. These data indicated that reduced protective role of Bifidobacterium in UC could be reversed by Pingkui enema. Although we did not investigate the effects Pingkui enema on the other probiotics, we just speculate that there might be other probiotics upregulated by Pingkui enema. Our present data at least support that regulation of gut probiotics may be another mechanism of therapeutic effects of Pingkui enema on TNBS-induced UC. Thirdly, previous study has shown that Bifidobacterium can repair the impaired intestinal epithelial barrier function and reduce the secretion of TNF-*α* and IFN-gamma in intestinal mucosa [40], suggesting the linkage of intestinal microbiota, gut epithelial barrier, and inflammatory reaction. Our present study demonstrated Pingkui enema improved epithelial barrier function (lower serum levels of *D*-LA and DAO). This effect of Pingkui enema might be the secondary consequence after regulation of gut probiotics (Bifidobacterium), which may partially contribute to the anti-inflammatory effects of Pingkui enema. It should be noted that whether there is other mechanism of the anti-inflammatory effects of Pingkui enema, such as its antioxidant property, or direct suppression of the transcriptional pathways of cytokines needs further investigation.

At present, fecal bacteria transplantation has been used to treat some diseases in which intestinal flora lose its balance and has achieved some curative effects [41]. However, concerns are raised that fecal bacteria transplantation can only temporarily change the composition of intestinal flora in UC patients and achieve temporary clinical remission but cannot continuously get clinical remission, and how to achieve long-term effective or remission by increasing the number of fecal bacteria transplantations needs further research to confirm [42]. By deeply exploring and advancing TCM therapy, novel strategies could be anticipated in the future for clinical UC patients.

## 5. Conclusions

Our present study shows that Chinese herbal enema alleviates TNBS-induced UC, which might be via promoting the colonization of Bifidobacterium in the intestinal tract, regulation of inflammatory cytokine, and protection of intestine epithelial barrier.

## Figures and Tables

**Table 1:** Composition of Pingkui enema.

Chinese name	Plant name	English name	Amount (g)	Place of origin
Qin Pi	*Fraxinus rhynchophylla* Hance	Fraxini cortex	20	Zhe Jiang, China
Huang Bo	*Phellodendron amurense* Rupr.	Phellodendri chinensis cortex	20	Si Chuan, China
Bai Touweng	*Pulsatilla chinensis* (Bunge) Regel	Radix pulsatillae	20	Si Chuan, China
Huang Lian	*Coptis chinensis* Franch.	Rhizoma Coptidis	10	Si Chuan, China
Xian Hecao	*Agrimonia eupatoria* L.	Hairyvein Agrimony	30	Yun Nan, China
San Qi	*Panax pseudoginseng* var. *notoginseng* (Burkill) G.Hoo and C.L.Tseng	Panax Notoginseng	3	Yun Nan, China
Zi Cao	*Lithospermum officinale* var. *erythrorhizon* (Siebold and Zucc.) Maxim.	Radix Arnebiae	15	Liao Ning, China
Bai Ji	*Bletilla striata* (Thunb.) Rchb. f.	Rhizoma Bletillae	10	Gui Zhou, China

**Table 2:** Evaluation of disease activity index.

Symptom	Score			
	0	1	2	3
Increase of stool times	0	1-2	3-4	>5
Gross bleeding	Negative	Occasionally	Often	All
Mucosa inflammation (colonoscopy)	Negative	Mild	Moderate	Severe
Weigh loss (%)	0	1–5	6–10	11–15

**Table 3:** Evaluation of pathological score. The total score was the sum of epithelial score and inflammatory cell infiltration.

Score	Epithelial cells	Inflammatory cell infiltration
0	Normal form	No infiltration
1	Goblet cell loss	Infiltration in the basal layer of crypt
2	Large area loss of goblet cells	Infiltration reaches the mucosal muscle layer
3	Crypt cells loss	Infiltration deep into the mucosal muscle layer, accompanied by mucosal thickening and edema
4	Large area loss of crypt cells	Infiltration to the submucosa

## Data Availability

Data used to support the findings of the study are available on request.

## Abbreviations

UC: Ulcerative colitis
TCM: Traditional Chinese medicine
TNBS: 2,4,6-Trinitrobenzene sulfonic acid
RT-PCR: Real-time fluorescence quantitative polymerase chain reaction
IL-8: Interleukin-8
IL-13: Interleukin-13
TNF-*α*: Tumor necrosis factor-alpha
ELISA: Enzyme-linked immunosorbent assay
FMT: Fecal bacteria transplantation
IBD: Inflammatory bowel disease.
